# The positive and negative consequences of stressors during early life

**DOI:** 10.1016/j.earlhumdev.2015.08.008

**Published:** 2015-11

**Authors:** Pat Monaghan, Mark F. Haussmann

**Affiliations:** a Institute of Biodiversity, Animal Health and Comparative Medicine, College of Medical, Veterinary and Life Sciences, University of Glasgow, Graham Kerr Building, Glasgow G12 8QQ, UK; b Department of Biology, College of Liberal Arts and Sciences, Bucknell University, Lewisburg, PA 17837 USA

**Keywords:** Glucocorticoids, Corticosterone, Ageing, Longevity, Telomeres, Hormesis

## Abstract

We discuss the long-term effects of stress exposure in pre- and early postnal life. We present an evolutionary framework within which such effects can be viewed, and describe how the outcomes might vary with species life histories. We focus on stressors that induce increases in glucocorticoid hormones and discuss the advantages of an experimental approach. We describe a number of studies demonstrating how exposure to these hormones in early life can influence stress responsiveness and have substantial long-term, negative consequences for adult longevity. We also describe how early life exposure to mild levels of stressors can have beneficial effects on resilience to stress in later life, and discuss how the balance of costs and benefits is likely dependent on the nature of the adult environment.

## 1 Introduction

Very few animals live in an environment that does not change in time and space. For most, day turns to night, seasons come and go, and habitats differ from one place to the next. Such changes are largely predictable. Animals are generally well adapted to deal with this kind of environmental variation, having evolved biological rhythms and movement patterns that maximise their success in passing on their genes to the next generation. Other kinds of environmental change are less predictable—the weather and food supplies fluctuate, predator and competitor numbers vary, and social pressures change. Facing unpredictable, and potentially dangerous, episodic change is more challenging. To deal with these threats, animals have evolved a suite of stress responses that can be turned on when the challenge appears, inducing changes in physiology and behaviour that maximise survival, and turned off again when the challenge has passed. In vertebrates, the main endocrine system that allows animals to cope with unpredictable change is the hypothalamic–pituitary–adrenal (HPA) axis. Perceived stressors activate the HPA axis, resulting in hormonal changes, largely involving the glucocorticoid hormones, which turn off currently non-essential activities and stimulate others. The animal enters a so-called ‘emergency state’ [1], in which activities such as growth, body maintenance, and reproduction are suspended, and energy is directed towards counteracting and surviving the imminent danger. This prioritisation is obviously adaptive, but if growth and body maintenance are suspended for long periods, this can be damaging over the long term, potentially increasing disease risk and the pace of degeneration in later life [2].

Stress responses are therefore both a friend and foe, and the costs and benefits are balanced to give the best fitness outcome. The extent to which long-term costs matter in an evolutionary sense varies among species. Evolution through natural selection has shaped the life history traits of animals (age and size at maturity, patterns of growth, number of offspring, relative investment in self maintenance) to maximise their lifetime reproductive success. For some species, growing fast and then having a single major reproductive event followed by death gives the best fitness outcome, while for other species, the best strategy is growing slowly, living a long time and repeatedly producing small numbers of offspring. For the former life history strategy, costs that are incurred over the long term are less important to fitness than is the case for the latter strategy. We should therefore expect animals with different life histories to have evolved different coping strategies. Furthermore, the probability of encountering environmental stressors is likely to vary in both time and space since some periods and places are safer than others. Therefore, any information that the developing animals can obtain on the prevailing environmental circumstances they may live in will enable the phenotype to be adjusted to better prepare them to cope with what they are most likely to encounter. This information on their future environment may calibrate their stress responses in a manner that maximises Darwinian fitness benefits (i.e. lifetime reproductive output). Hence we expect some environmental shaping of the stress responses.

It is only relatively recently that developmental biologists have come to appreciate that the environment is not simply permissive of development but can also instruct it. As a result, a new discipline has emerged, termed ecological developmental biology, or Eco-Devo [3]. While genes set the potential range of phenotypes possible, we now know that the pattern of gene expression is influenced by environmental inputs and that these can span generations. A distinction is drawn in the Eco-Devo literature between direct and indirect environmental effects both of which can affect the developing animal. In the former case, the environment directly affects the developing individuals, and in the latter case, it is through environmental effects on the physiology or actions of the parent(s), most usually the mother. In species with high levels of pre- and postnatal parental care, developing offspring are buffered against some direct environmental effects; birds and mammals, for example, regulate the developmental temperature of their offspring within narrow limits. Other factors, however, do affect the developing offspring. The level of exposure to stressors in early life, in particular, when the architecture of the body is being determined, may be crucial in calibrating the developing animal's stress response system. These types of beneficial adjustments of phenotypic traits based on signals transmitted directly or indirectly to the developing animals have been termed ‘predictive adaptive responses’ (PARS) [4]. This relies on some environmental stability—that is the conditions experienced during development need to be predictive of subsequent conditions over an appropriate time frame. It is not clear whether the apparent pathologies associated with early stress exposure [5] are in fact a consequence of phenotypic adjustments that make the best of bad situation or represent stress levels outside of the evolved coping capabilities. An additional complexity is added in that, at least for some stressors, exposure to mild levels in early life gives rise to an enhanced resilience to stress that can be advantageous in later life. This positive effect of stress exposure is termed hormesis and is discussed further below in Section 4.

In this mini-review, we discuss stress exposure in both prenatal and early postnatal life within the evolutionary framework outlined above. We describe a number of relevant experiments that we have carried out on the consequences of early life stress exposure over differing time scales. The experiments that we describe have been carried out mainly in birds, for a number of reasons. Birds are endothermic vertebrates which, like mammals, maintain their developing embryos at an optimal temperature during development; *in ovo* in the bird, *in utero* in the mammal. Birds and mammals have a high level of parental care, and nutrients are supplied prenatally by the mother to the embryo via the egg yolk or the placenta. Like primates, birds are long-lived, iteroparous breeders, having relatively slow reproductive rates (e.g. compared with salmon or mice), slow senescence rates, and long lives. Importantly, however, that the avian embryo develops within a sealed system makes it possible to experimentally separate effects due to the pre- and postnatal environment and to separate direct and indirect environmental effects.

We do not provide a fully comprehensive review of the literature in this extensive, multidisciplinary field. Rather, we discuss a number of key issues that can be profitably viewed in an evolutionary framework. We focus on stressors that induce increases in glucocorticoid hormones, such as increased predation risk or social stress. In an experimental context, the increase in glucocorticoids can be stimulated by presenting the stressor itself or via direct hormonal manipulations. While early life stress exposure can have a large number of consequences, we emphasise here the effects on telomere dynamics during growth and development, which thereby have the potential to produce long-term effects on health and longevity. Telomeres are specialised areas of non-coding DNA and associated proteins that occur at the ends of the chromosomes. In vertebrates, the telomeres comprise tandem repeats of the nucleic acid sequence TTAGGG, and the very end of the telomere is folded back on itself to form a loop. Telomeres enable the cell machinery to recognise these looped chromosome ends and also protect the coding sequences from the loss during cell division. This loss occurs because, during DNA replication, the very end of the lagging DNA strand is not completely replicated. Additional telomeric sequence can be lost because of oxidative damage to the nucleotides, which interrupts replication; the high guanine content of telomeres appears to make them particularly vulnerable to oxidative damage. Once telomeres become critically short, their function is impaired and, under normal circumstances, cells cease dividing and either die or remain in an altered non-dividing state. Telomeres can be restored. In most animals, including vertebrates, this involves the enzyme telomerase, which is variably active in different tissue types. In many long-lived vertebrates, including humans and many long-lived birds, telomerase is downregulated in most somatic cells, thus limiting the cells' replicative potential. The resulting cell loss, and the accumulation of senescent cells, are thought to contribute to a decline in tissue and organ function with age. Following this, telomere length and loss have been shown to relate to survival [6], [7], and several studies have found that early life telomere length is the best predictor of longevity. What determines early life telomere length, which has a significant heritable component and involves direct and indirect environmental effects, is difficult to disentangle. An increasing body of literature from both animal and human work is demonstrating that stress exposure in early life can increase telomere loss, and suggests that this is linked to glucocorticoid exposure [7], [8]. Stress experienced by parents not only affects their own health but can also have long-lasting repercussions for their offspring. This can come about because the offspring experience the same adverse environment that is affecting their parents, or indirectly because of the environmental conditions that the parents provide, either pre- or postnatally. To better understand these connections, an experimental approach is critical, in which, for example, effects due to the individual's early and adult environment, or the parent and offspring environment, can be teased apart. Longitudinal studies that follow individuals across their life course are also important so that identifying long-term outcomes for individuals, and age-related changes, is not confounded by certain phenotypes being more or less likely to survive to old age. Such studies are very difficult to do in humans.

## 2 Prenatal effects

Several human and animal studies have established links between stressful conditions during embryonic and foetal development and disease risk later in life. The well-documented effects that appeared in children born during the Dutch ‘Hunger winter’ famine at the end of World War II illustrate how serious and long lasting these effects can be. Women who were pregnant during the famine experienced severe nutritional and psychological stress, and the children they gave birth to had increased incidence of disease decades after the famine had ended [9]. While some of these effects were likely to have been caused by foetal malnutrition, overexposure to glucocorticoids also seems to have been involved. These two causes are of course interconnected.

Stress experienced by mothers can expose embryos to maternally derived glucocorticoids through the placenta in mammals and through their presence in eggs of oviparous species [10]. The transfer of maternal glucocorticoids to offspring might in part be an inevitable and detrimental result of poor environmental conditions. Indeed, prenatal stress often has effects reminiscent of the protracted and repeated stress exposure mentioned in the introduction, including reduced birth weights and growth rates, compromised immunity and increased disease rates, and reduced survival [2]. However, if the mother can strategically control the transfer of glucocorticoids, maternal glucocorticoids might benefit offspring by serving as a maternally mediated cue that alters offspring phenotype in preparation for a stressful environment. For example, in both birds and mammals, maternally derived glucocorticoids can alter the responsiveness of the HPA axis, thereby influencing how offspring respond to stressful situations later in life. Being more stress reactive may be beneficial in a dangerous environment. Understanding the extent to which changes in reactivity are adaptive, or the outcome a physiological constraint imposed by system design, is an active area of research and will depend in part on a particular species life history strategy [11].

Stress in the prenatal environment has been widely studied in mammals [5], [12], where elevated maternal glucocorticoids can permanently modify the development and subsequent function of the HPA axis in offspring. The direction of this modification is variable and depends not only on the timing, duration, and magnitude of glucocorticoid exposure but also varies by species and sex [12]. For example, in guinea pigs (*Cavia porcellus*), a single exposure to a 48-hr period of maternal stress resulted in male offspring with reduced baseline and stress-induced HPA activity, but females from the same litter exhibited elevated baseline and stress-induced HPA activity. In another guinea pig study, adult male offspring born to mothers exposed to stress on days 50–52 of gestation exhibited elevated baseline glucocorticoids, while those born to mothers exposed to stress on gestational days 60–62 exhibited normal baseline levels, but heightened stress-induced HPA activity. These studies highlight the complex nature of HPA programming by maternal glucocorticoids, which influence the developing foetus by binding to specific glucocorticoid receptors. These processes are probably best understood in the rat model, where maternal glucocorticoids in developing rats reduce the number of glucocorticoid type I and II receptors in the hippocampus [5]. This reduction results in impaired negative feedback control of glucocorticoid secretion, which often produces higher baseline levels and a prolonged duration of the stress response. In two separate correlative studies on humans, mothers that reported experiencing higher levels of stress during pregnancy produced offspring with shorter telomeres at birth [13] and in young adulthood [14]. These human studies are necessarily correlative and include potential biases associated with self-reporting, which makes it difficult to determine causal relationships. Nevertheless, they establish an interesting pattern that invites further study using experimental models.

Studying the effects of prenatal stress exposure in an oviparous system has the advantage of allowing the characterisation of embryonic responses to increased glucocorticoid exposure independent of maternal responses during embryo development. One of the inherent issues with studying embryonic responses to glucocorticoids in a placental system is that it is nearly impossible to increase embryonic exposure to glucocorticoids without also altering maternal physiology. Therefore, it is difficult to isolate the direct response of embryos to glucocorticoids from indirect responses induced via changes in maternal physiology. Additionally, an oviparous system has the added benefit of easier access to the embryo, making manipulations of the embryonic endocrine environment much simpler. In particular, avian oviparous systems are particularly attractive because the avian neuroendocrine system is very similar to the mammalian system and thus responds in similar ways to stressors. In addition, the extra embryonic membranes in birds have remarkably similar functions to the extra embryonic membranes that form part of the placenta and umbilical cords in mammals. Perhaps most importantly, the two major mechanistic hypotheses thought to underlie the association between maternal stress and postnatal effects mentioned above (foetal malnutrition and overexposure to glucocorticoids) are extremely difficult to manipulate independently in mammals but can be done effectively in an oviparous model.

Even with these advantages, there have been relatively few studies exploring the effects of prenatal glucocorticoid exposure on the development of the HPA axis in birds. Work in Japanese quail (*Coturnix japonica*) demonstrates that there can be complex sex-specific and developmental stage-specific effects of prenatal glucocorticoid exposure on subsequent stress responses [15]. Additionally, a recent study in domestic chickens (*Gallus domesticus*) involved direct manipulation of corticosterone levels, the primary avian glucocorticoid, in eggs. Juvenile chickens that had been exposed to experimentally increased corticosterone *in ovo* had a protracted decline in corticosterone after exposure to a stressor compared to control juveniles. This experimentally induced increase in exposure to corticosterone gave rise to higher levels of oxidative stress and an over-representation of short telomeres compared to the control birds [16]. In this experiment, differences in postnatal care among individuals were controlled for because neither control nor experimental chicks received any parental care, possible in this highly precocial species. Therefore, these effects are due to exposure of corticosterone while *in ovo*.

## 3 Postnatal effects

The effects of stress exposure in early postnatal life have been experimentally studied in most detail in rats. Stressors have included maternal separation, which gives rise to increased activity of the HPA axis and reduced cognitive performance later in life. Repeated early handling by humans, on the other hand, or high levels of maternal grooming reduce stress sensitivity and improve aspects of cognitive performance. In humans, harsh conditions in childhood are also associated with increased stress reactivity [17]. How this links to long-term longevity has, however, been little studied in an experimental setting in mammals.

A recent experiment carried out in zebra finches (*Taeniopygia guttata* maximum lifespan in captivity ca 9 years, less in the wild) demonstrates that there can be substantial lifespan penalties associated with increased exposure to glucocorticoids. The corticosterone levels of chicks were increased via a daily dose of corticosterone (within the natural range) between 12 and 28 days after hatching, which resulted in increased reactivity of the HPA axis. A heightened stress responsiveness, similar to that seen in the prenatal work in chickens and quail, and postnatal work in rats, was still detectable when the birds were young adults. In addition, the birds were then all kept in similar conditions and their longevity monitored over a 3-year period. The early life stress exposure had no effect on survival of the birds as chicks, juveniles, or young adults or on their reproductive performance. However, the effect on longevity was striking with those birds having been exposed to stress in early life showing a faster rate of ageing and substantially reduced longevity; by the end of 3 years, around 85% of the control birds were still alive compared with only just over 65% of those whose stress hormone exposure in early life was increased [18]. A key aspect of this study is that it was experimental—the animals were randomly allocated to the different treatments, and after the early life hormone manipulation, they all lived in the same, benign, environmental conditions. This is important because there could be a number of potentially confounding factors in non-experimental studies, such as a positive co-variation between the early and later life environment being stressful, the exposure to early life stress not being random with some individuals being more likely to experience such stressors. These factors are almost impossible to control in studies in humans. This zebra finch study illustrates that early life stress exposure can have substantial long-term consequences; though whether these give the best fitness outcome might also depend on the later life environment (see Section 4 below).

A similar effect to that described in zebra finches was found in a field study using chicks of the European shag, a long-lived seabird (*Phalacrocorax aristotelis*, maximum lifespan in the wild over 30 years). In this study, we also examined telomere length. The increased exposure to glucocorticoids gave rise to increased telomere loss during growth in these shag chicks, even though growth itself was not affected by the experimental treatment [19]. This suggests that increased telomere loss could be involved in the long-term effects of early stress exposure on ageing and longevity. Socially induced stress in early life can also have long-term consequences for physiology, behaviour, and telomere length. For example, in a study using wild European starlings (*Sturnus vulgaris*, maximum recorded lifespan in the wild 23 years), two chicks from broods of four were moved to foster broods such that they were then larger than, and dominant, over their nest mates, and their two siblings were moved to other broods such that they were smaller than, and subordinate to, their nest mates; brood size in the foster nests was held constant at 4 chicks. While social position did not affect growth, being a subordinate chick is known to be stressful. We found that the chicks put into a subordinate position had more telomere loss and hence shorter telomeres at the end of growth than their biological siblings that had been placed in brood where they were the dominant chicks [20]. In a similar study in which social stress was manipulated in starling nestlings by manipulating brood size, chicks that were at the bottom of the brood hierarchy showed more telomere loss, an effect that was still evident when the birds were fully grown and independent [21]. The behaviour of these birds was examined when they were adults (6–14 months old); those that had shown more telomere loss in early life (between days 4 and 55) showed more impulsive behaviour, that is, they were unwilling to wait for larger rewards, preferring a small reward now rather than a delayed but larger reward [22]. The results of these studies, which involve direct effects of environmental conditions on many phenotypic traits, show that early life social stress can influence later life behaviour and induce potential reduced longevity as indicated by increased telomere loss. Whether these behavioural changes represent an adaptive response to a changing and stressful environment is not known, but possible.

## 4 Hormetic effects

As mentioned in the introduction, an optimally functioning stress response system is essential to survival—not responding to imminent danger is likely to lead to an early death. As shown in 2, 3, the level of stress exposure in early life can influence the sensitivity of the HPA axis. A number of experimental studies have demonstrated that exposure to a mild form of a stressor in early life can mean that the response to stressors in adult life is more effective, a process termed hormesis. But, why have this form of environmentally determined calibration? The explanation might involve the advantages of minimising unnecessary exposure to glucocorticoid hormones. Being highly reactive in a challenging environment is beneficial, but if the environment is not harsh or dangerous, more moderate responses may be appropriate. This has clearly been demonstrated in an experiment in the zebra finch. Some birds were exposed to short episodes of a mild level of heat stress in early life while others were not exposed to heat stress. Later in life, some birds from each group experienced a somewhat stronger level of the stressor again, while others in each group did not. The amount of oxidative damage that occurred when the stressor was experienced in adulthood was reduced in the group that had the early life priming, demonstrating their improved coping ability. Interestingly however, when longevity was examined, those birds that had experienced the stressor in early life, but did not experience it again later in life, showed lower longevity than any other group[23]. Under these circumstances, having a mismatch between what the animal prepares itself for, and what it then experiences, is costly. It is possible therefore that the survival effects shown in the zebra finch experiment discussed in Section 3 might have been different had the adult environment been more stressful. Such environmental mismatches are the basis of the ‘thrifty phenotype’ explanation of the poor health associated with poor nutrition in early life. If early life conditions induces a ‘storage phenotype’ and this is followed by access to high-calorie food later, then obesity and problems associated with metabolic syndrome can occur [4].

## 5 Conclusions

Understanding the extent to which the developmental histories of individuals influence their ability to cope with environmental challenges is an important area of research that spans diverse disciplines and many levels of biological enquiry. We need to be able to link an animal's current phenotype with its individual history. Experimental studies in birds and mammals, and correlative studies in humans, show that early life conditions have substantial long-term consequences for individuals, and potentially also for their offspring. The sensitivity of the HPA axis appears to be calibrated by early life experience, and this is associated with substantial consequences for behaviour, telomere loss, and longevity. We need to understand how and why organisms react in particular ways to early life adversity, and whether these responses represent adaptive adjustments where benefits outweigh costs, or can be rendered harmful due to environmental mismatches. An evolutionary perspective, in addition to mechanistic studies, has an important contribution to make to such research. Working with, rather than fighting against, our evolutionary history is more likely to reap benefits.

## Research directions

There are many areas of investigation of the effects of early life adversity that remain to be explored. Here, we focussed on stress exposure, the HPA axis, and longevity. However, other stressors commonly encountered include those related to variability in food supply during development, including the long-term effects of catch-up growth, which could also involve the HPA axis and telomere dynamics. A particularly important question is whether responses to stress in early life are different from those when stress is experienced in adult life. Are changes more permanent when they occur during development? Are there critical windows when stress reactivity is permanently altered? When is stress beneficial, and are hormetic effects transferrable across stressor types? It is also important to clarify our understanding of whether the phenotypic adjustments we see to physiology and behaviour are adaptive or not, and whether they vary with species life histories and environmental variability.

## Conflict of interest statement

The authors declare no conflict of interest.
